# Comparison of the effectiveness of two adjustable negative pressure ureteral access sheaths combined with flex ureteroscopy for ≤ 2 cm renal stones

**DOI:** 10.1038/s41598-024-55333-w

**Published:** 2024-02-27

**Authors:** Deheng Cui, Qinghong Ma, Shengbiao Xie, Guangzhi Wang, Guanghai Li, Guoqiang Chen

**Affiliations:** Department of Urology, The Second Hospital of Longyan, Longyan, 364000 Fujian China

**Keywords:** Intelligent intrarenal pressure control platforms, Flexible ureteral access sheath, Retrograde intrarenal surgery, Negative pressure, Urology, Renal calculi

## Abstract

To compare the safety and effectiveness of the combination of intelligent intrarenal pressure control platforms (IPCP) and flexible ureteral access sheath (FUAS) combined with retrograde intrarenal surgery (RIRS) for the treatment of renal stones less than 2 cm. We retrospectively collected 383 patients with renal stones who underwent RIRS in our department from June 2022 to October 2023. Inclusion criteria: stone length or the sum of multiple stone lengths ≤ 2 cm. Finally, 99 cases were included and divided into an IPCP group (n = 40) and FUAS group (n = 59) based on surgical methods. The main endpoint was the stone-free rate (SFR) at third months after surgery, with no residual stones or stone fragments less than 2 mm defined as stone clearance. The secondary endpoints were surgical time and perioperative complications, including fever, sepsis, septic shock, and perirenal hematoma. There was no statistically significant difference in general information between the two groups, including age, gender, body mass index, comorbidities, stone side, stone location, stone length, urine bacterial culture, and hydronephrosis. The operation time for IPCP group and FUAS group was 56.83 ± 21.33 vs 55.47 ± 19.69 min (p = 0.747). The SFR of IPCP group and FUAS group on the first postoperative day was 75.00% vs 91.50% (p = 0.024). The SFR was 90.00% vs 94.90% in the third month (p = 0.349).In IPCP group, there were 11 cases with stones located in the lower renal calyces and 17 cases in FUAS group. The SFR of the two groups on the first day and third months after surgery were 45.50% vs 88.20% (p = 0.014) and 63.60% vs 94.10% (p = 0.040), respectively, with statistical differences. For kidney stones ≤ 2 cm, there was no difference in SFR and the incidence of infection-related complications between IPCP and FUAS combined with RIRS, both of which were superior to T-RIRS. For lower renal caliceal stones, FUAS has a higher SFR compared to IPCP.

## Introduction

Renal stones are a common benign disease of the urinary system, with an incidence rate of 5.8% among Chinese adults^1^. There are many treatment methods for kidney stones, such as active monitoring, medication lithotripsy, extracorporeal shock wave lithotripsy (ESWL), retrograde intrarenal surgery (RIRS), and percutaneous nephrolithotripsy (PCNL). For renal stones smaller than 2 cm, the stone-free rate (SFR) of RIRS is better than that of ESWL, and the complications are less than those of PCNL^2^. RIRS through the natural lumen of the human body, without adding new wounds, and has the advantages of fast recovery and less pain. Of course, many drawbacks limit the further promotion of RIRS. Traditional flexible ureteroscopy and ureteral access sheath (UAS) have poor water circulation, and blurred vision, and often rely on postoperative self-removal of stones, which limits the effectiveness of stone removal^3^. In addition, perioperative infection complications of traditional RIRS (T-RIRS) such as fever, sepsis, and septic shock have been reported to have an incidence rate of up to 37%^4^. The close correlation between intrarenal pressure and infection has long been confirmed^5,6^.

In recent years, many innovative devices have emerged to reduce the incidence of infection, among which negative pressure suction technology is rapidly being promoted in China. At present, commonly used devices include intelligent intrarenal pressure control platforms (IPCP) and flexible UAS(FUAS) at the head end, which have their respective advantages. Still, there is little research on this aspect^7^. Our centre had already proficiently implemented these two technologies. This study aimed to compare the safety and effectiveness of the combination of IPCP and FUAS combined with RIRS for the treatment of renal stones less than 2 cm, to provide better treatment plans for patients.

## Method

We retrospective collected 383 patients with renal stones who underwent RIRS in our department from June 2022 to October 2023. Inclusion criteria: stone length or the sum of multiple stone lengths ≤ 2 cm. Exclusion criteria: ① preoperative double J tube placement, ② uncontrolled urinary tract infection, ③ ureteral stricture, ④ severe cardiopulmonary insufficiency, ⑤ renal insufficiency, solitary kidney, ⑥ bilateral RIRS, ⑦ uncontrolled hemorrhagic disease. Finally, 99 cases were included and divided into an IPCP group (n = 40) and FUAS group (n = 59) based on surgical methods. Both groups of patients underwent preoperative biochemical, coagulation tests, urine routine, urine bacterial culture, kidney ureter bladder plain film (KUB), and non-contrast enhanced computer tomography (NCCT) of the urinary system. The data of two groups were analysed, such as age, gender, stone burden, blood creatinine, comorbidity, urine culture, and perioperative complications. Patients with positive preoperative urine cultures would be treated with sensitive antibiotics based on drug sensitivity results until the urine culture turns negative. Patients with negative preoperative urine cultures received a single dose of antibiotics before surgery to prevent infection. Each group of surgeries was completed by the same experienced surgeon who specializes in stones.

This study was approved by the Ethics Committee of the Second Hospital of Longyan City, Fujian Province, and informed consent was obtained from all patients. Our study was conducted by the ethical standards of the 1964 Declaration of Helsinki and its subsequent amendments.

### Observation indicators and evaluation criteria

The main endpoint was the SFR in the third month after surgery, with no residual stones or stone fragments less than 2 mm defined as stone clearance^8,9^. The secondary endpoints were surgical time and perioperative complications, including fever, sepsis, septic shock, and perirenal hematoma. The operation time was calculated as the insertion of UAS until the double J tube was inserted. The diameter of a stone refers to the longest diameter of a single stone or the sum of the lengths of multiple stones measured on NCCT. NCCT of the urinary system was performed on the first day and three months after surgery, and the double J tube was removed one month after surgery.

### Surgical method

#### IPCP group

**Figure 1:**
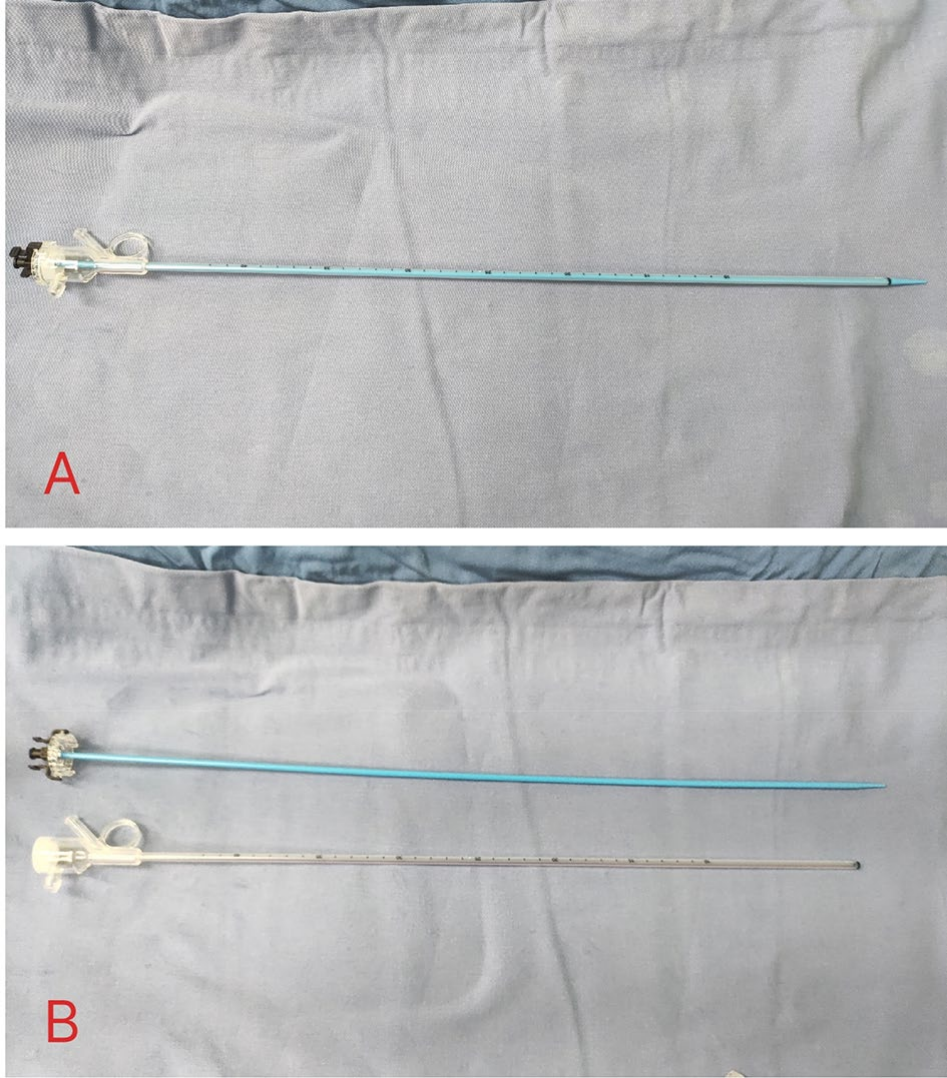
Ureteral access sheath of intelligent intrarenal pressure control platforms. Combination of the sheath and inner core (**A**); Separation of the sheath and inner core (**B**).

**Figure 2:**
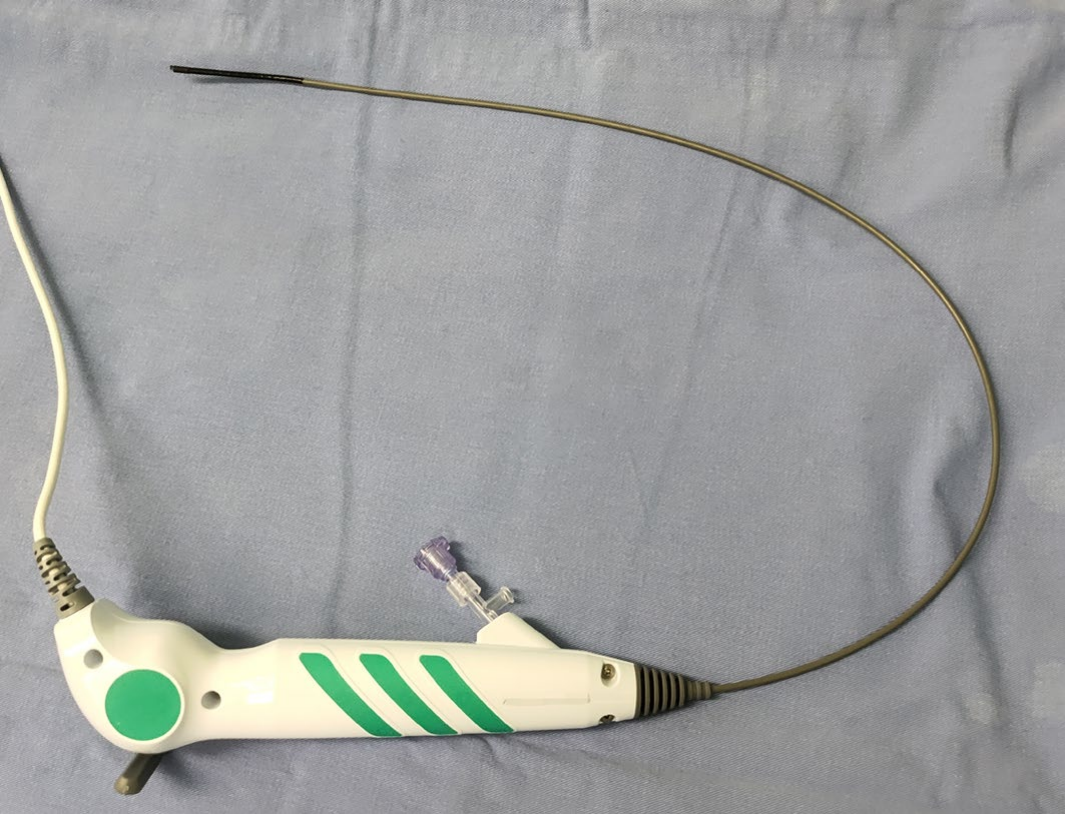
A disposable flex ureteroscope (Zebra).

All patients received general anesthesia and were in the oblique supine position. The ureter was examined by a ureteroscope and a COOK guide wire was inserted into the renal calyces. The pressure sensor and pipeline were correctly connected, the integrity of each pipeline was checked, and the pressure monitoring was calibrated. The pressure warning value in the renal pelvis was set to 30 mmHg, and the perfusion flow rate was 50-150 ml/min. The tail of UAS was connected to negative pressure suction, and the attraction was set to 0.02–0.04Mpa. The head end of the 12F UAS (Fig. 1) was placed at the junction of the renal pelvis and ureter, and a disposable ureteroscope (Zebra, Fig. 2) combined with 200 μm holmium laser fibre was used for crushed stone, with energy setting of 0.6–0.8 J and frequency setting of 20–30 Hz. A vortex was generated in the renal calyx by using the flushing solution to remove as many stone fragments as possible. Finally, a 6F double J tube and a 16F catheter were placed.

#### FUAS group

**Figure 3:**
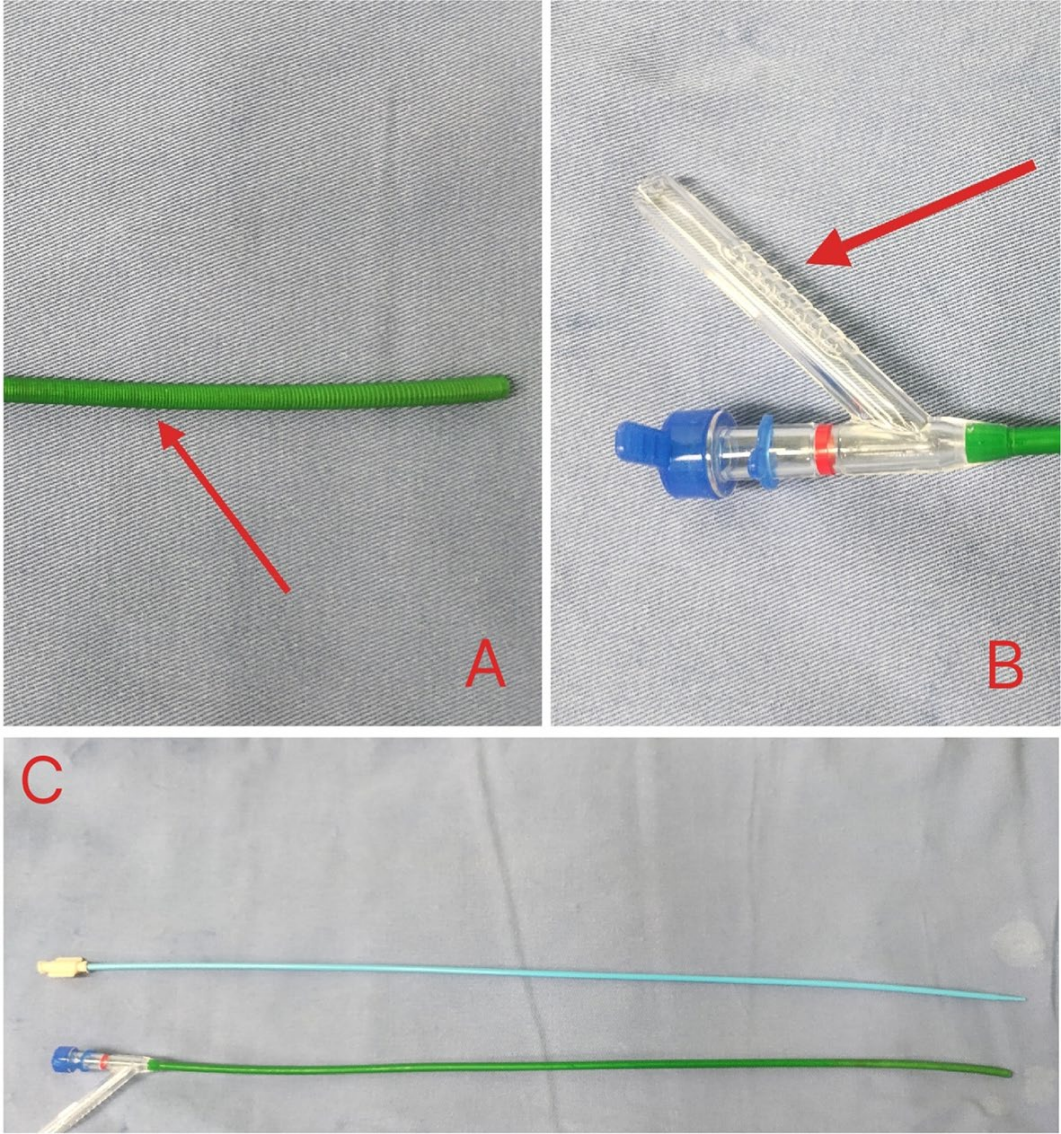
The bendable head of FUAS (**A**), Connecting the tail end of negative pressure suction (**B**), sheath and inner core of FUAS (**C**). *FUAS* flexible ureteral access sheath.

All patients received general anesthesia and were in the oblique supine position. The ureter was examined by a ureteroscope and a COOK guide wire was inserted into the renal calyces. The 12F FUAS (Xin Kangshun, Fig. 3) was inserted into the renal pelvis along the guide wire. The tail of FUAS was connected to negative pressure suction, and the attraction was set to 0.02–0.04Mpa. Disposable ureteroscopy combined with 200 μm holmium laser fibre was used for lithotripsy, with an energy setting of 0.6–0.8 J, frequency setting of 20-30 Hz, and perfusion flow rate of 50–150 ml/min. The flexible head of FUAS could enter almost all renal calyces and remove stone fragments. Samely, A 6F double J tube and a 16F catheter were placed.

### Statistical method

Firstly, the normality and homogeneity tests of variance were performed on continuous data. If it followed a normal distribution and had homogeneity of variance, two independent samples were selected for the t-test, and the results were expressed as mean ± standard deviation. Contrary, the Mann–Whitney *U*-test was chosen, and the results were represented by the median (lower quartile to upper quartile). The counting data was represented by an example (%). Grade count data was tested using the Mann–Whitney U test, while non-grade data was tested using χ^2^ inspection. *P* < 0.05 was considered statistically significant. All statistical analyses were conducted using commercially available software SPSS 27.0.

## Result

There was no statistically significant difference in general information between the two groups, including age, gender, BMI, comorbidities, stone side, stone location, stone length, urine bacterial culture, and hydronephrosis. The operation time for IPCP group and FUAS group was 56.83 ± 21.33 vs 55.47 ± 19.69 min (*p* = 0.747), with no statistical difference (Table 1). The SFR of IPCP group and FUAS group on the first postoperative day was 75.00% vs 91.50% (*p* = 0.024), indicating a statistically significant difference. The SFR was 90.00% vs 94.90% in the third month (*p* = 0.349), with no statistically significant difference (Table 2).

**Table 1:** General information of the two groups.

Variable	IPCP group	FUAS group	*P* value
Age (year)	53.50 [44.50,62.25]	55.00 [45.00,63.00]	0.437
Gender, n (%)			0.630
Male	23 (57.50)	37 (62.70)	
Female	17 (42.50)	22 (37.30)	
BMI (kg/m^2^)	23.45 ± 3.03	24.29 ± 2.88	0.165
Urine culture n (%)			0.074
Positive	2 (5.00)	10 (16.90)	
Negative	38 (95.00)	13 (83.10)	
Comorbidities			0.972
None	34 (85.00)	50 (84.70)	
Diabetes	6 (15.00)	9 (15.30)	
Stone site, n (%)			0.161
Right	22 (55.00)	24 (40.70)	
Left	18 (45.00)	35 (59.30)	
Stone location			0.887
Lower calyx	11 (27.50)	17 (28.80)	
Others	29 (72.50)	42 (71.20)	
Stone diameter (mm)	13 [11.00,15.00]	14 [11.00,16.00]	0.100
Stone CT density (HU)			0.252
≤ 1000	25 (62.50)	30 (50.80)	
> 1000	15 (37.50)	29 (49.20)	
Hydronephrosis			0.291
None	15 (37.50)	25 (42.40)	
≤ 1cm	14 (35.00)	20 (33.90)	
1–2cm	8 (20.00)	5 (8.50)	
> 2cm	3 (7.50)	9 (15.30)	

*BMI* body mass index, *HU* hounsfield units.

**Table 2:** Perioperative complications and postoperative effect evaluation of two groups.

Variable	IPCP group	FUAS group	*P*
Mean operative time (min)	56.83 ± 21.33	55.47 ± 19.69	0.747
Fever, n (%)	1 (2.50)	0	0.222
Spesis, n (%)	1 (2.50)	2 (3.40)	0.800
Urinary leakage, n (%)	1 (2.50)	0	0.222
Total SFR at first day, (%)	75.00	91.50	0.024*
Total SFR at third month, (%)	90.00	94.9	0.349
SFR of lower calyx at first day, (%)	45.50	88.20	0.014*
SFR of lower calyx at third month, (%)	63.60	94.10	0.040*

*SFR* stone-free rate.

*p < 0.05, statistically significant difference.

In IPCP group, there were 11 cases with stones located in the lower renal calyces and 17 cases in FUAS group. The SFR of the two groups on the first day and third months after surgery were 45.50% vs 88.20% (*p* = 0.014) and 63.60% vs 94.10% (*p* = 0.040), respectively, with statistical differences. In addition, there was 1 case of fever, 1 case of sepsis, and 1 case of urine extravasation in IPCP group after surgery. There were 2 cases of sepsis in FUAS group, without fever or urine leakage. There were no patients with perirenal hematoma, renal artery embolism, septic shock, or death in both groups.

## Discussion

In T-RIRS, difficulty in perfusion fluid circulation can easily lead to renal pelvis hypertension. When the pressure in the renal pelvis is higher than 30 mmHg, the risk of infection will be significantly increased^10^. The combination of RIRS and negative pressure technology has various advantages, such as maintaining low pelvic pressure, accelerating stone removal speed, maintaining a clear view, and reducing complications, especially infections. During the surgery, the stones are directly removed from the renal, reducing the risk of postoperative failure to expel stones and improving SFR. The intelligent control platform was originally invented and clinically applied by Professor Leming Song, and its safety and effectiveness have been confirmed^3,7^. This study compared and analyzed the effectiveness and safety of two techniques combined with RIRS in the treatment of renal stones smaller than 2 cm, and shared our surgical experience.

The SFR on the first day after surgery in FUAS group was higher than that in IPCP group, with a statistically significant difference. However, there was no statistically significant difference in the SFR at 3rd month. We analysed that the reasons for the differences may be related to the following factors: ① In the FUAS group, the head of the drone can touch or approach the stones, which can be quickly removed and easy to operate. ② IPCP group relied on the vortex currents generated by the flushing solution to flush out the stones. The significantly dilated renal pelvis and slender renal calyces made it more difficult to create a vortex, increasing the difficulty of stone removal. Another key fact to remember is that both technologies rely on the flex scope to exit outward while the water flows outward to expel the stone fragments, rather than relying on increasing negative pressure suction. The SFR of T-RIRS for renal stones ≤ 2 cm was approximately 85.7%, which was lower than the SFR of each group in our study^11,12^. During T-RIRS, fragments were rarely removed and often required self-removal of stones after surgery. However, recent studies had shown that even fragments smaller than 2 mm were at risk of failure in stone removal^13^.

The incidence of urosepsis after surgery in both groups was 2.5% vs 3.4% (p = 0.800), both lower than the 5% incidence of T-RIRS^14,15^. Reducing the incidence of urosepsis could improve the safety of RIRS while maintaining low renal pelvic pressure during operation played a crucial role^10,16^. The two devices had different usage techniques, and our experience was as follows: ① IPCP monitored the pressure in the renal pelvis, and the pressure was controlled within a safe range. In addition, while maintaining low pressure, the fragment was removed through the pressure difference of rapid circulation of high-flow liquid^7^. ② The FUAS group did not have pressure monitoring equipment during operation, while the negative pressure state of the renal pelvis could also be maintained through the following characteristics. First. The renal pelvis and calyces were slightly invaginated rather than full. Second, if the field of view suddenly became cloudy, the device needed to be checked for malfunctions. Negative pressure equipment may also carry certain risks. When IPCP is blocked, it can automatically stop infusion, while FUAS will continue to pump flushing fluid into the renal pelvis, causing a sudden increase in pressure. A brief increase in pelvic pressure may also increase the risk of urosepsis.

**Figure 4:**
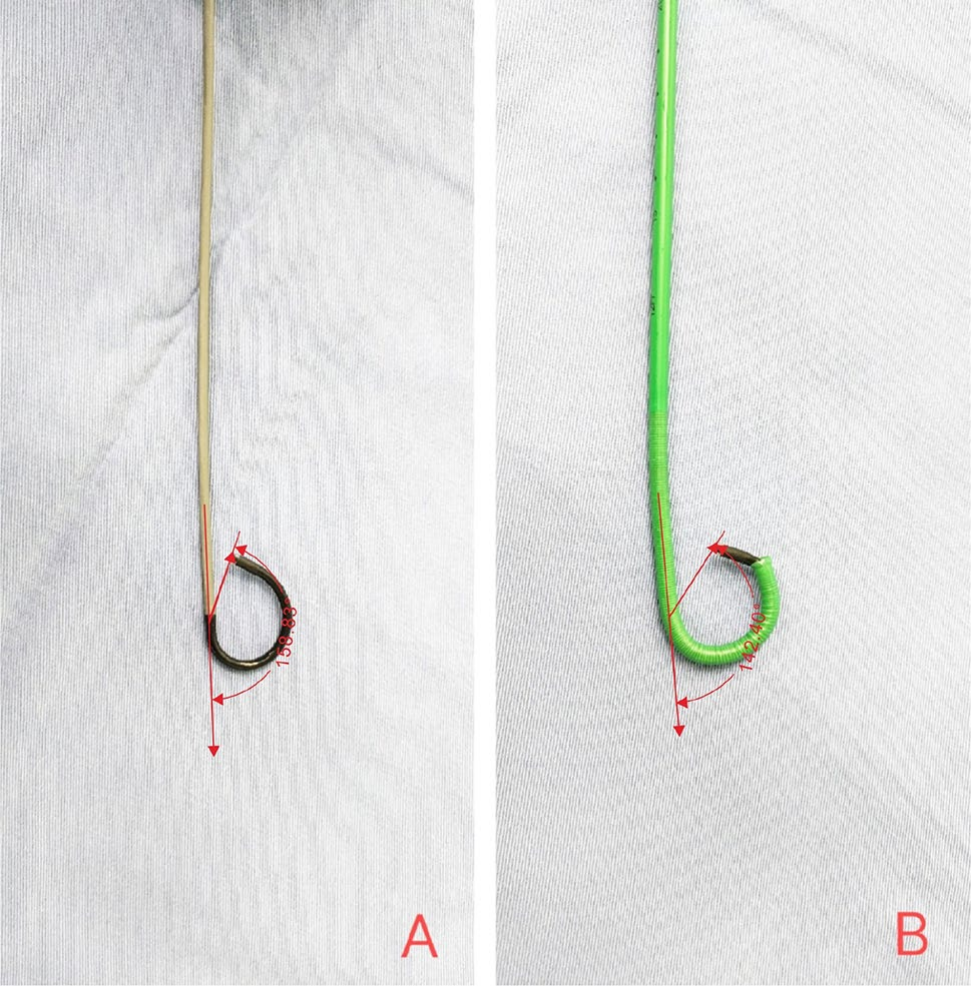
The maximum degree of downward bending of a disposable flex ureteroscope (**A**), the maximum degree of downward bending of a disposable flex ureteroscope combined with flexible ureteral access sheath (**B**).

For low renal caliceal stones, the SFR in IPCP group was significantly lower than that in FUAS group, at 63.60% vs 94.10% (*p* = 0.040). The end of UAS in IPCP group was located in the pyeloureteral junction, which was the same as traditional UAS and had no advantage in handling lower renal caliceal stones. On the contrary, the head of FUAS could be bent arbitrarily. When the flex ureteroscope was inserted into FUAS, the downward curvature of the flexible ureteroscope decreased from 158.83° to 142.40°, only 16.43° (Fig. 4). The head of FUAS entered the lower renal calyx along the ureteroscope, and the stone was immediately removed. In our study, there were fewer patients with simple lower renal caliceal stones, and the conclusion may had some bias. In the future, more samples will be needed to confirm this conclusion.

Our study was retrospective and not randomized and prospective, therefore, selection bias was difficult to avoid. The study was a single-centre retrospective study with a limited sample size, which may have resulted in a lack of confidence in the statistical analysis of the data. A larger, prospective, multi-center randomized controlled trial with standardized patient selection and a broader participant base will be conducted in the future.

## Conclusion

For kidney stones ≤ 2 cm, there was no difference in SFR and the incidence of infection-related complications between IPCP and FUAS combined with RIRS, both of which were superior to T-RIRS. For lower renal caliceal stones, FUAS has a higher SFR compared to IPCP.

### Supplementary Information

Supplementary Table S1.

## Data Availability

All data generated or analysed during this study are included in this published article and its supplementary information files. Supplementary Table S1 contains information on the collection methods for each sample.
